# Drug-specific risk and characteristics of lupus and vasculitis-like events in patients with rheumatoid arthritis treated with TNFi: results from BSRBR-RA

**DOI:** 10.1136/rmdopen-2016-000314

**Published:** 2017-01-17

**Authors:** Meghna Jani, William G Dixon, Lianne Kersley-Fleet, Ian N Bruce, Hector Chinoy, Anne Barton, Mark Lunt, Kath Watson, BSRBR-RA*, BSRBR-RA Control Centre Consortium*, Deborah P Symmons, Kimme L Hyrich

**Affiliations:** 1 Arthritis Research UK Centre for Epidemiology, Institute of Inflammation and Repair, University of Manchester, Manchester Academic Health Science Centre, Manchester, UK; 2 Centre for Musculoskeletal Research, Institute of Inflammation and Repair, University of Manchester, Manchester Academic Health Science Centre, Manchester, UK; 3 National Institute of Health Research Manchester Musculoskeletal Biomedical Research Unit, Central Manchester Foundation Trust and University of Manchester, Manchester Academic Health Science Centre, Manchester, UK

**Keywords:** Anti-TNF, DMARDs (synthetic), Epidemiology, DMARDs (biologic), Rheumatoid Arthritis

## Abstract

**Objective:**

To compare the risk of lupus-like events (LLEs) and vasculitis-like events (VLEs) in tumour necrosis factor-α inhibitor (TNFi)-treated patients with rheumatoid arthritis (RA) to those receiving non-biological disease-modifying antirheumatic drugs (nbDMARDs).

**Methods:**

Patients were recruited to the British Society for Rheumatology Biologics Register—RA, a national prospective cohort study. Two cohorts recruited between 2001 and 2015: (1) patients starting first TNFi (adalimumab, etanercept, infliximab and certolizumab) (n=12 937) and (2) biological-naïve comparison cohort receiving nbDMARDs (n=3673). The risk of an event was compared between the two cohorts using Cox proportional-hazard models, adjusted using propensity scores. Rates of LLE/VLE were compared between TNFi and nbDMARD patients.

**Results:**

The crude incidence rates for LLEs were: TNFi 10/10 000 patient-years (pyrs) (95% CI 8 to 13) and nbDMARD 2/10 000 pyrs (95% CI 1 to 6); for VLEs: TNFi 15/10 000 pyrs (95% CI 12 to 19) and nbDMARD 7/10 000 pyrs (95% CI 4 to 12). The risk of both events was highest in the first year of TNFi treatment. After adjusting for differences in baseline characteristics, there was no difference in risk of LLEs (_adj_HR 1.86; 95% CI 0.52 to 6.58) or VLEs (_adj_HR 1.27; 95% CI 0.40 to 4.04) for TNFi compared to nbDMARD-treated patients. Infliximab conferred the highest overall risk, followed by etanercept, although 95% CIs overlapped following adjustment.

**Conclusions:**

In one of the largest biological registers, the absolute risk of both events is low. The addition of TNFi to nbDMARD does not alter the risk of either event in patients with RA selected for TNFi. This is the first study to assess the risk of these outcomes in a prospective, observational cohort.

**Key messages**

**What is already known about this subject?**

- Lupus-like events (LLEs) and vasculitis-like events (VLEs) have been reported in association with tumour necrosis factor-α inhibitor (TNFi) therapy from spontaneous pharmacovigilance; however, the actual risk from prospective observational studies has not been studied.

**What does this study add?**

- This study from BSRBR-RA showed that the absolute risk of both events was low in the TNFi group (LLE 10/10 000 patient-years; VLE 15/10 000 patient-years) and after adjusting for baseline differences, there was no increased risk of LLE/VLE in TNFi-treated patients compared to TNFi-naïve patients.

**How might this impact on clinical practice?**

- The results provide reassurance to patients that such events are rare in TNFi and TNFi-naïve patients.
- High-disease activity was associated with higher rates, while concomitant treatment, such as sulfasalazine use, was associated with lower rates of LLE/VLE, highlighting the importance of adequate control of RA joint disease to potentially minimise risk of such events.

## Introduction

Tumour necrosis factor-α inhibitor (TNFi) for the treatment of rheumatoid arthritis (RA) has been associated with asymptomatic immunological alterations to autoimmune pathology with systemic manifestations. A single case of lupus-like event (LLE) was reported following two infusions of infliximab in the first TNFi randomised controlled trial.^1^ The number and spectrum of autoimmune diseases reported to be induced by TNFi agents have increased in parallel with their widespread use. The most commonly described events are LLEs and vasculitis-like events (VLEs).^2^

Induction of antinuclear antibodies (ANAs) following TNFis is well recognised, with a small proportion of patients developing immune-mediated adverse events (AEs), including LLE and VLEs.^3^ Seroconversion to ANA positivity has been associated with secondary non-response to TNFi treatment,^3^ ^4^ and to the development of antidrug antibodies or immunogenicity^5^ with monoclonal antibody TNFi drugs (infliximab/adalimumab). Immunogenicity has itself also been linked to a diverse range of VLEs with varying degrees of severity, from limited cutaneous involvement to life-threatening systemic manifestations.^6^ ^7^

Most data on LLEs and VLEs are derived from spontaneous pharmacovigilance, case reports and retrospective studies.^8^ ^9^ While published data suggest a drug-specific difference in ANA seroconversion,^3^ reports to date do not enable robust comparison between drugs in terms of actual clinical AEs. Furthermore, it is not known if factors, such as concomitant non-biological disease-modifying antirheumatic drugs (nbDMARDs), attenuate the risk of these events, although their use is associated with reduced immunogenicity in TNFi-treated patients.^10^

The primary aim of this study was to determine the incidence of LLE and VLE in patients with RA treated with TNFi, compared to biological-naïve patients treated with nbDMARDs. Additional objectives were to: (1) evaluate drug-specific risk, (2) determine the risk of each outcome following exclusion of putative causes and (3) identify factors associated with each outcome.

## Patients and methods

### Participants

The British Society for Rheumatology Biologics Register—RA (BSRBR-RA) is a UK-based national, observational, prospective cohort study established in 2001, which aims to study the long-term safety of biological treatment. Patients starting treatment with TNFi (infliximab, etanercept, adalimumab and certolizumab) are enrolled for observational follow-up. As per the UK national guidelines, patients are eligible for TNFi if they have active disease (disease activity score of 28 joints (DAS28) >5.1), despite treatment with at least two nbDMARDs, one of which should be methotrexate.^11^ A comparison cohort of biological-naïve patients with RA, with active disease (defined as a DAS28 score of ≥4.2), despite current treatment with nbDMARDs, was recruited in parallel between 2002 and 2009. Ethical approval for BSRBR-RA was granted in December 2000 by the North West Multicentre Research Ethics Committee (reference 00/8/53). Written consent was obtained from all participants recruited.

### Baseline assessment

Baseline demographic and clinical information was collected via clinician questionnaires. Consultants were specifically asked if the patient had a history of systemic vasculitis and nailfold vasculitis prior to starting a biologic. Other comorbidities, such as systemic lupus erythematosus (SLE), were recorded via a free text box. Self-reported ethnicity was captured within the baseline patient questionnaire. Patients were requested to complete a Stanford Health Assessment Questionnaire (HAQ).^12^

### Follow-up

Follow-up information, including medication changes and AEs, were captured via three routes: clinician-completed questionnaires (6-monthly for 3 years then annually), patient diaries (6-monthly for first 3 years only) and flagging by the Office for National Statistics who notify the register in the event of death or cancer. All reports of serious events are follow-up with the hospital to request further information. For LLE, this was via a disease-specific pro-forma; for VLE, this was via open correspondence.

### Case definition and verification of AEs

We used all relevant MedDRA codes and free text searches within the AE reported fields (see online supplementary table S4) to identify potential cases. All possible reported events of either LLE or VLE were reviewed in detail using information provided to the register. The types of LLE events described in previous literature from France, USA and Spain have been heterogeneous ranging from dsDNA-positive patients with cutaneous lupus to fewer ‘full blown’ lupus meeting ACR classification criteria.^2^ ^8^ ^13^ It has been argued that LLE associated with TNFi therapy is a distinct syndrome compared to SLE.^14^ Since classification criteria for drug-induced lupus does not currently exist, the Dubois' guidelines for drug-induced lupus were used^15^ to provide a more sensitive definition. The full guidelines are outlined in online supplementary table S1. Briefly, this includes continuous treatment with a lupus-inducing drug for ≥1 month, common or multisystem presenting symptoms consistent with lupus, laboratory profile consistent with lupus and improvement of symptoms following drug cessation. Patients were classified as having an LLE if clinically identified as such and ≥2 criteria were met. Following verification (performed by MJ), LLE cases were additionally classified according to standard SLE classification criteria (1997 ACR^16^ and 2012 SLICC criteria^17^) for descriptive purposes only. A single set of classification criteria could not be applied due to the heterogeneous characteristics of vasculitis events, ranging from cutaneous to multisystem manifestations. Therefore, such reports were flagged as such by the reporting physician; verification was performed clinically following additional requests for clinical information where necessary.

10.1136/rmdopen-2016-000314.supp1 supplementary data

### Statistical analysis

Patients with a physician diagnosis of RA, who had at least one clinician-completed follow-up form by 31 May 2015, were included. The primary outcome was the first verified LLE or first verified VLE per participant following the start of TNFi drug (or registration for the nbDMARD cohort). For the LLE analysis, patients with known SLE overlap recorded at baseline were excluded. Patients were excluded from the analysis if they had known systemic vasculitis at baseline. Events were attributed to TNFi if they occurred when the patient was receiving TNFi or within 90 days of the first missed dose. Follow-up time was censored at event of interest, death, drug stop date (plus 90 days) or last physician follow-up, whichever came first. Only time on a first TNFi was included to best attribute any drug-specific risk. In the nbDMARD cohort, follow-up time was censored if the patient switched to a biological drug.

Crude incidence rates were presented as events per 10 000 patient-years (pyrs). Cox proportional-hazard models were used to compare event rates between TNFi and nbDMARD cohorts. Cumulative hazards were compared between the different drugs using Nelson-Aalen plots. A flexible parametric spline model was used to model time-varying incidence rates in the TNFi cohort. Adjustment for differences between cohorts was made using propensity scores using inverse probability of treatment weights (IPTW; see online supplementary material). Variables were identified a priori and included age, gender, disease duration, HAQ score, baseline DAS28; rheumatoid factor status, recruitment year, baseline nbDMARD use, baseline oral steroid use and ethnicity (the latter variable for the LLE analysis only). Comorbidity was included as a composite variable generated from the presence of: ischaemic heart disease (myocardial infarction and/or angina); stroke, hypertension, chronic lung disease (asthma, bronchitis or emphysema), renal disease, liver disease, diabetes mellitus or depression as described previously.^18^ Ethnicity was stratified as white and non-white for analyses (table 1). Missing data were replaced using multiple imputation to avoid the bias induced by a complete case analysis (see online supplementary table S2).

**Table 1:** Baseline characteristics of nbDMARD and TNFi-treated patients

				First TNFi drug				
	nbDMARD (n=3673)	All TNFi (n=12 937)	p Value*	Etanercept (n=4516)	Adalimumab (n=4362)	Infliximab (n=3363)	Certolizumab (n=696)	p Value†
*Demographic features*								
Age, mean (SD) years	60 (12)	56 (12)	<0.001	56 (12)	57 (12)	56 (12)	56 (12)	0.012
Gender, % female	72	76	<0.001	77	76	76	76	0.62
Rheumatoid factor-positive, %	2135 (58)	8199 (63)	<0.001	2883 (64)	2688 (62)	2250 (67)	378 (58)	<0.001
Ethnicity								
White	2952 (80)	10 467 (81)	<0.001	3638 (81)	3588 (83)	2795 (83)	446 (64)	
Black	24 (0.6)	86 (0.7)		28 (0.6)	34 (0.8)	19 (0.6)	5 (0.7)	0.42
South Asian	32 (0.9)	228 (2)		82 (2)	73 (2)	65 (2)	8 (1)	
Chinese	2 (0.1)	31 (0.2)		9 (0.2)	12 (0.3)	9 (0.2)	1 (0.2)	
Other	15 (0.4)	117 (0.9)		36 (0.8)	31 (0.7)	41 (1.2)	9 (1)	
Not recorded	648 (18)	2002 (16)		723 (16)	624 (14)	428 (13)	227 (32)	
Smoking history, n (%)								
Current smoker	869 (24)	2762 (21)	<0.001	911 (20)	980 (23)	734 (22)	137 (20)	<0.001
Former smoker	1452 (40)	4888 (38)		1728 (38)	1660 (38)	1274 (38)	226 (33)	
Never smoked	1334 (36)	5159 (40)		1830 (41)	1683 (38)	1338 (40)	308 (44)	
Not recorded	18 (0.5)	128 (1)		47 (1)	39 (1)	17 (0.5)	25 (3)	
Disease duration, median (IQR)	6 (1–15)	10 (5–18)	<0.001	11 (5–19)	10 (5–18)	12 (6–19)	5 (2–12)	<0.001
DAS28 score, mean (SD)	5.1 (1.3)	6.5 (1)	<0.001	6.5 (1)	6.4 (1)	6.6 (1)	5.9 (1)	<0.001
HAQ score, mean (SD)	1.5 (0.8)	2.0 (0.6)	<0.001	2.0 (0.6)	1.9 (0.6)	2.1 (0.6)	1.5 (0.8)	<0.001
Comorbidity, n (%)								
None	1544 (42)	6031 (47)	<0.001	2026 (45)	2060 (47)	1582 (47)	363 (52)	0.001
1 comorbidity	1270 (35)	4397 (34)		1522 (34)	1476 (34)	1177 (35)	222 (32)	
2 comorbidity	596 (16)	1839 (14)		701 (15)	601 (14)	457 (14)	80 (11)	
≥3 comorbidities	263 (7)	670 (5)		267 (6)	225 (5)	147 (4)	31 (5)	
*Treatment-related factors*								
Number of prior nbDMARDS, median (IQR)	2 (1–3)	3 (3–5)	<0.001	4 (3–5)	3 (2–4)	4 (3–5)	3 (2–3)	<0.001
On methotrexate, n (%)	2449 (67)	7932 (61)	<0.001	1921 (43)	2554 (59)	2983 (89)	474 (68)	<0.001
On sulfasalazine, n (%)	1245 (34)	2395 (18)	<0.001	682 (15)	971 (22)	513 (15)	229 (33)	<0.001
On leflunomide, n (%)	495 (13)	1182 (9)	<0.001	377 (8)	495 (11)	244 (7)	66 (9)	<0.001
On azathioprine, n (%)	93 (2)	309 (2)	0.62	123 (3)	91 (2)	92 (3)	3 (0.4)	0.001
On minocycline, n (%)	1 (0.03)	18 (0.1)	0.08	5 (0.1)	7 (0.2)	6 (0.2)	0	0.63
On hydroxychloroquine, n (%)	639 (17)	1842 (14)	<0.001	519 (11)	716 (16)	348 (10)	259 (37)	<0.001
Baseline steroid use, n (%)	836 (23)	5348 (41)	<0.001	2018 (45)	1617 (37)	1541 (46)	172 (25)	<0.001

*p Value represents the significance of differences between the nbDMARD and TNFi cohorts using χ^2^ tests for categorical outcomes and Wilcoxon rank sum tests for continuous variables.

†p Value represents the significance of differences between the four TNFi drugs using χ^2^ tests for categorical outcomes and Kruskal-Wallis rank tests for continuous variables. Values <1 are represented up to one decimal point.

DAS28, 28 joint count Disease Activity Score; HAQ, Health Assessment Questionnaire; nbDMARDs, non-biological disease-modifying antirheumatic drugs; TNFi, tumour necrosis factor-α inhibitor.

Two sensitivity analyses were performed. For the LLE analysis, patients on drugs known to be associated with drug-induced lupus at baseline (see online supplementary table S3) and the use of sulfasalazine, leflunomide, minocycline and penicillamine were excluded.^15^ ^19^ ^20^ For the VLE analysis, patients who had a probable secondary vasculitis aetiology (eg, infection at the time of event), known baseline nailfold vasculitis, on other medications associated with VLEs at baseline (see online supplementary table S3) or use of minocycline and penicillamine during the study period were excluded.^21^ All analysis was performed using Stata V.13.0 (StataCorp, College Station, Texas, USA).

## Results

### Baseline characteristics

A total of 3673 nbDMARD and 12 937 TNFi-treated patients were included (see online supplementary figure S1). The TNFi cohort was older, had proportionally more women with higher disease severity (table 1). Differences between patients treated with infliximab, etanercept and adalimumab were less marked while patients treated with certolizumab (registered after 2010) had shorter disease duration and less severe disease at initiation.

### Event rates and hazard estimates

**Figure 1:**
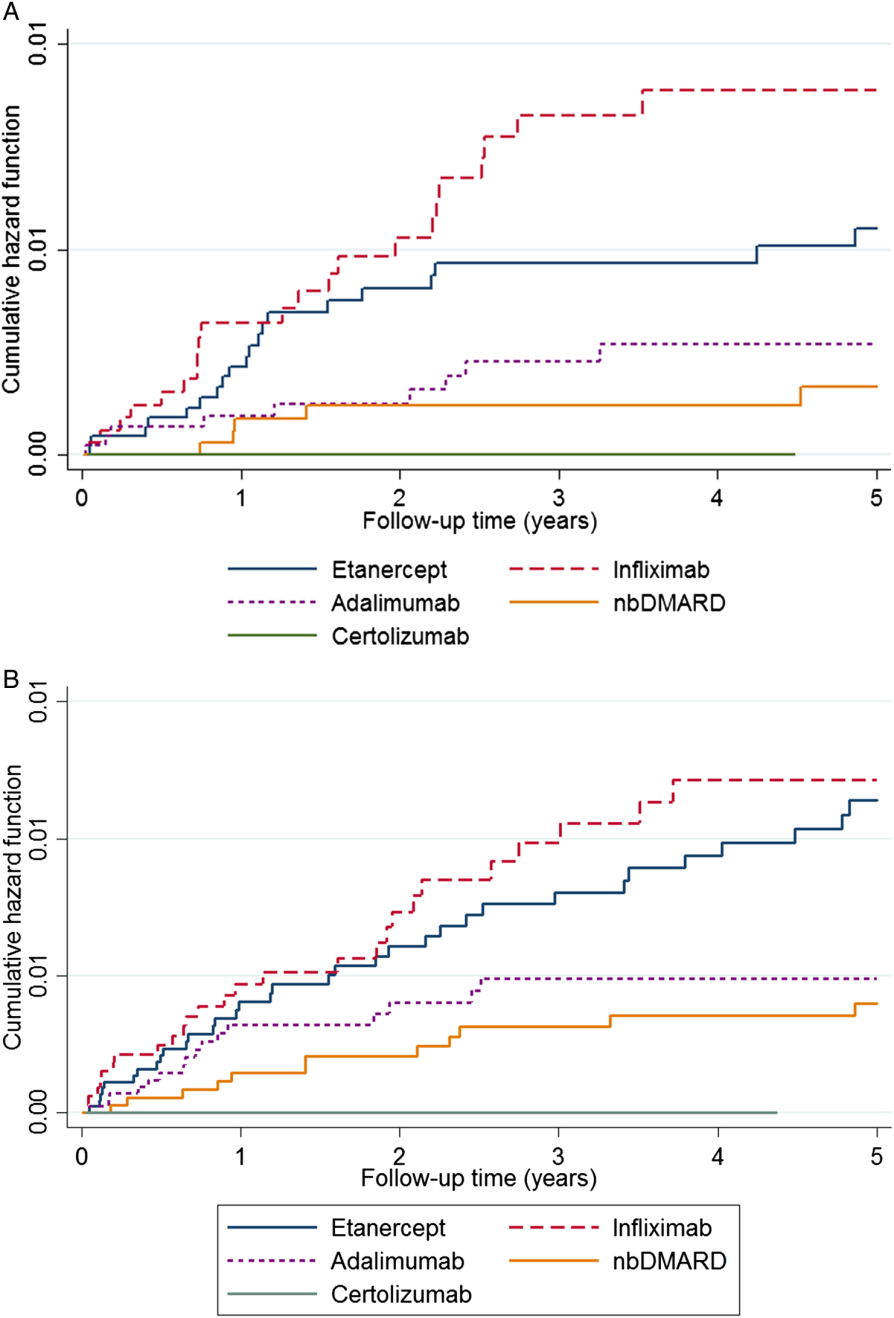
Nelson-Aalen plots comparing nbDMARD and TNFi cohorts for each outcome. (A) Lupus-like events; (B) Vasculitis-like events. Cumulative hazard estimates are demonstrated using Nelson-Aalen plots for each drug evaluated in the register. Infliximab appears to have the highest risk in both analyses, followed by etanercept and adalimumab. nbDMARD, non-biological disease-modifying antirheumatic drug.

There were 59 LLEs (54 TNFi and 5 nbDMARD). The incidence of LLE was 10/10 000 pyrs (95% CI 8 to 13) in the TNFi cohort compared to 2/10 000 pyrs (95% CI 1 to 6) in the nbDMARD cohort. After full adjustment, the risk of LLE in TNFi-treated patients compared to nbDMARDs was no longer significant (_adj_HR 1.86; 95% CI 0.52 to 6.58) (table 2). The risk appeared highest for infliximab (figure 1). Female gender, non-white ethnicity, baseline DAS28 score, baseline HAQ score and minocycline use were associated with increased risk of LLE, while baseline and concomitant sulfasalazine use were associated with lower rates (table 3). Following exclusion of patients on drugs known to be associated with drug-induced lupus, the _adj_HR was 2.33 (95% CI 0.56 to 9.71) (see online supplementary table S3).

**Table 2:** Crude incidence rates and HRs for lupus/vasculitis type events in nbDMARD and TNFi-treated patients (on drug+90 days analysis)

	nbDMARD (n=3673)	All TNFi (n=12 937)	Etanercept (n=4516)	Adalimumab (n=4362)	Infliximab (n=3363)	Certolizumab (n=696)
Lupus-like events						
Total follow-up time (patient-years)	20 815	53 159	21 595	17 343	13 181	1040
Follow-up per subject, median (IQR)	6.5 (3.4–9.0)	5.1 (2.0–8.9)	6.3 (2.5–10.0)	5.3 (2.2–8.0)	4.2 (1.6–8.4)	1.5 (0.8–2.6)
Number	5	54	20	11	23	0
Crude incidence rate of lupus-like event per 10 000 person-years (95% CI)	2.4 (1.0 to 5.7)	10.2 (7.8 to 13.2)	9.3 (6.0 to 14.3)	6.3 (3.5 to 11.5)	17.5 (11.6 to 26.3)	–
Unadjusted HR (95% CI)	Referent	3.93 (1.57 to 9.83)*	3.83 (1.44 to 10.21)*	2.40 (0.83 to 6.92)	6.59 (2.50 to 17.36)*	–
Age and gender adjusted (95% CI)	Referent	3.72 (1.47 to 9.35)*	3.60 (1.34 to 9.64)*	2.26 (0.78 to 6.53)	6.28 (2.38 to 16.61)*	–
Propensity score adjusted HR (95% CI)†	Referent	1.86 (0.52 to 6.58)	1.41 (0.41 to 4.90)	1.77 (0.33 to 9.36)	2.65 (0.75 to 9.35)	–
	n=3640	n=12 745	n=4450	n=4312	n=3292	n=691
Vasculitis-like events						
Total follow-up time (patient-years)	20 635	52 428	21 320	17 172	12 903	1033
Follow-up per subject; median (IQR)	6.5 (3.4–9.0)	5.1 (2.0–8.9)	6.3 (2.5–10.0)	5.3 (2.2–8.0)	4.2 (1.6–8.5)	1.5 (0.8–2.6)
Number of vasculitis-like events, n	14	81	37	18	26	0
Crude incidence rate of vasculitis-like events per 10 000 person-years (95% CI)	6.8 (4.0 to 12.4)	15.5 (12.4 to 19.2)	17.4 (12.6 to 24.0)	10.5 (6.6 to 16.6)	20.2 (13.7 to 29.6)	–
Unadjusted HR (95% CI)	Referent	2.12 (1.20 to 3.74)*	2.58 (1.39 to 4.77)*	1.38 (0.69 to 2.78)	2.70 (1.41 to 5.18)*	–
Age- and gender-adjusted HR (95% CI)	Referent	2.31 (1.30 to 4.09)*	2.84 (1.52 to 5.28)*	1.50 (0.74 to 3.03)	2.93 (1.52 to 5.64)*	–
Propensity score-adjusted HR (95% CI)‡	Referent	1.27 (0.40 to 4.04)	1.72 (0.53 to 5.57)	0.71 (0.21 to 2.47)	1.55 (0.46 to 5.20)	–

*p<0.05.

†Adjusted for age, gender, disease duration, baseline DAS28 score, baseline HAQ score, comorbidities, rheumatoid factor-positive status, year of recruitment, baseline steroid use, baseline nbDMARD use and ethnicity.

‡Adjusted for age, gender, disease duration, baseline DAS28 score, baseline HAQ score, comorbidities, rheumatoid factor-positive status, year of recruitment, baseline steroid and nbDMARD use.

DAS28, 28 joint count Disease Activity Score; HAQ, Health Assessment Questionnaire; nbDMARDs, non-biological disease-modifying antirheumatic drugs; TNFi, tumour necrosis factor-α inhibitor.

**Table 3:** Univariate analysis of individual covariates of immune-mediated adverse event risk and risk of TNFi in association with event

	HR (95% CI) for covariate	HR (95% CI) for anti-TNF agent
**Lupus-like events**		
Unadjusted		3.88 (1.54 to 9.23)
Age (per year)	0.98 (0.97 to 1.01)	3.79 (1.51 to 9.53)
Gender (male referent)	2.74 (1.25 to 6.00)*	3.83 (1.53 to 9.59)
Ethnicity (non-white referent)	2.90 (1.24 to 6.75)*	3.36 (1.34 to 8.43)
Rheumatoid factor-positive	1.08 (0.75 to 1.55)	3.91 (1.56 to 9.80)
Smoking (current smoking referent)	0.96 (0.49 to 1.67)	3.95 (1.57 to 9.88)
Disease duration	1.01 (0.99 to 1.04)	3.89 (1.55 to 9.77)
Baseline DAS28	1.58 (1.25 to 1.98)*	2.38 (0.90 to 6.29)
Baseline HAQ score	1.69 (1.11 to 2.56)*	2.73 (1.07 to 6.97)
Comorbidities (nil referent)		
1 comorbidity	1.11 (0.65 to 1.91)	3.95 (1.58 to 9.90)
2 comorbidities	0.77 (0.34 to 1.75)	
≥3 comorbidities	1.17 (0.41 to 3.38)	
Methotrexate use†	0.91 (0.54 to 1.51)	3.92 (1.57 to 9.83)
Baseline methotrexate use	1.03 (0.62 to 1.72)	3.95 (1.58 to 9.88)
Sulfasalazine use†	0.30 (0.12 to 0.75)*	3.38 (1.34 to 8.51)
Baseline sulfasalazine use	0.36 (0.16 to 0.84)*	3.53 (1.41 to 8.87)
Leflunomide use†	0.73 (0.29 to 1.82)	3.92 (1.57 to 9.83)
Baseline leflunomide use	0.83 (0.33 to 2.01)	3.93 (1.56 to 9.84)
HCQ use†	1.30 (0.70 to 2.45)	4.10 (1.63 to 10.31)
Baseline HCQ use	1.57 (0.86 to 2.89)	4.02 (1.61 to 10.08)
Minocycline use†	11.20 (1.55 to 80.81)*	3.88 (1.55 to 9.72)
Baseline minocycline use	14.31 (1.98 to 103.18)*	3.88 (1.55 to 9.72)
On steroid at baseline	0.83 (0.49 to 1.36)	4.23 (1.68 to 10.62)
**Vasculitis-like event**		
Unadjusted		2.12 (1.20 to 3.74)
Age	1.01 (0.98 to 1.03)	2.27 (1.28 to 4.03)
Gender (male referent)	0.67 (0.46 to 1.0)	2.15 (1.22 to 3.80)
Ethnicity (non-white referent)	0.51 (0.12 to 2.08)	2.17 (1.18 to 4.00)
Rheumatoid factor-positive	1.82 (1.18 to 2.78)*	2.04 (1.16 to 3.61)
Current smoking	1.30 (0.86 to 1.98)	2.14 (1.21 to 3.79)
Disease duration	1.03 (1.01 to 1.04)*	2.16 (1.20 to 3.90)
DAS score	1.42 (1.20 to 1.68)*	1.44 (0.77 to 2.69)
HAQ score	1.65 (1.19 to 2.28)*	1.78 (0.93 to 3.42)
Comorbidities (nil referent)		
1 comorbidity	1.46 (0.98 to 2.19)	2.11 (1.19 to 3.72)
2 comorbidities	0.69 (0.35 to 1.38)	
≥3 comorbidities	1.16 (0.48 to 2.61)	
Methotrexate use†	0.68 (0.47 to 0.98)*	2.07 (1.17 to 3.67)
Baseline methotrexate use	0.79 (0.54 to 1.14)	2.07 (1.17 to 3.66)
Sulfasalazine use†	0.46 (0.29 to 0.82)*	1.84 (1.03 to 3.24)
Baseline sulfasalazine use	0.56 (0.33 to 0.97)*	1.96 (1.10 to 3.47)
Leflunomide use†	0.75 (0.38 to 1.48)	2.10 (1.19 to 3.72)
Baseline leflunomide use	0.61 (0.27 to 1.38)	2.08 (1.18 to 3.68)
HCQ use†	0.81 (0.46 to 1.41)	2.11 (1.19 to 3.74)
Baseline HCQ use	0.91 (0.51 to 1.62)	2.11 (1.19 to 3.72)
Combination nbDMARDs (≥2, including methotrexate)	0.66 (0.43 to 1.00)	2.05 (1.16 to 3.63)
On steroid at baseline	1.20 (0.82 to 1.75)	2.07 (1.16 to 3.67)

The association between candidate confounders and the outcome (first lupus/vasculitis-like event), irrespective of the treatment group. The final column reports the effect of each baseline covariate on the estimated treatment effect.

*p<0.05.

†Use of nbDMARD versus not use during the study period (assumes the risk returns to baseline as soon as the patient is off the drug).

Anti-TNF, tumour necrosis factor-α inhibitor; DAS28, 28 joint count Disease Activity Score; HAQ, Health Assessment Questionnaire; nbDMARDs, non-biological disease-modifying antirheumatic drugs.

There were 95 cases of VLEs (81 TNFi and 14 nbDMARD) giving an incidence rate for VLE in the TNFi cohort of 15/10 000 pyrs (95% CI 12 to 19) and 7/10 000 pyrs (95% CI 4 to 12) in the nbDMARD cohort. Following adjustment using IPTW, there was no significant difference in the risk of VLE for TNFi compared to nbDMARD-exposed patients (_adj_HR 1.27; 95% CI 0.40 to 4.04; table 2). Rheumatoid factor-positive status, disease duration, baseline DAS28 and HAQ scores were associated with a higher risk while baseline and concomitant sulfasalazine and concomitant methotrexate were associated with a lower risk (table 3). Exclusion of secondary causes leading to VLE demonstrated no significant differences between groups (_adj_HR 1.05; 95% 0.32 to 3.45; see online supplementary table S3).

**Figure 2:**
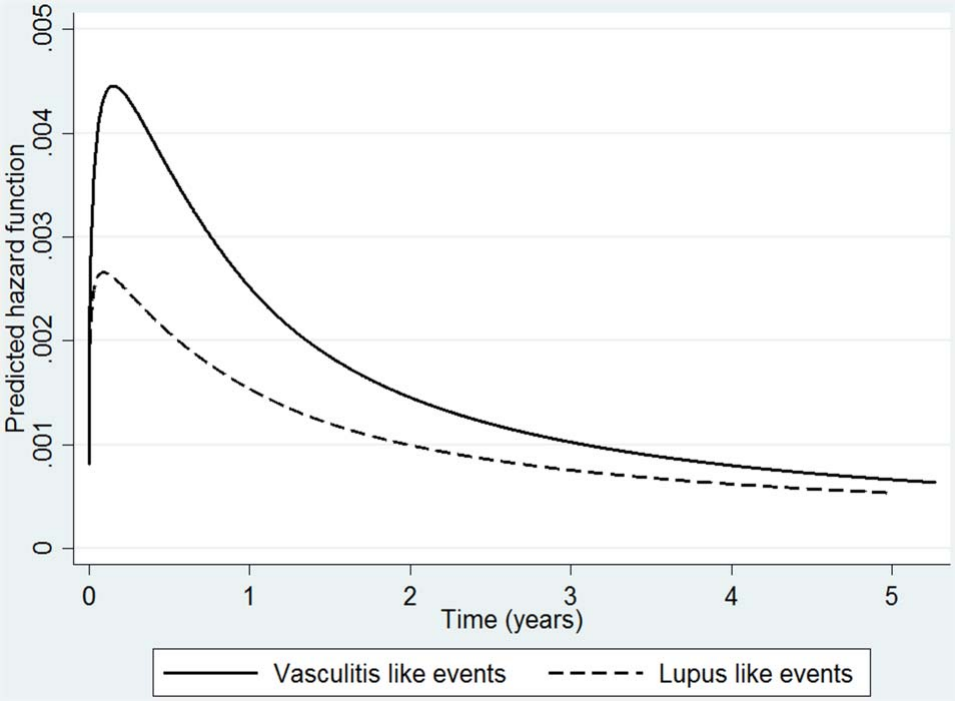
Spline model demonstrating time-varying risk of lupus and vasculitis events in the TNFi cohort. Hazard for lupus- and vasculitis-like events over time in TNFi cohort, using a flexible parametric spline model. TNFi, tumour necrosis factor-α inhibitor.

TNFi median time to first LLE was 1.2 years for TNFi patients (IQR 0.6–2.5) and 1.0 year (IQR 0.9–1.4) for nbDMARD patients. For VLEs, median time to event was 1 year (IQR 0.5–2.5) compared to 1.8 years (IQR 0.9–3.3) for TNFi and nbDMARD patients, respectively. The hazard for LLE and VLE in the TNFi-treated cohort was greatest in the early months of treatment (figure 2) then steadily decreased over the course of follow-up.

### Characteristics of events

For LLE, 48/54 (89%) patients exposed to anti-TNF had cutaneous involvement and was the sole manifestation (plus ANA-positive or consistent skin biopsy) in 30/54 (56%). Of the LLE cases, three patients were known to be ANA-positive at baseline with no SLE manifestations prior to treatment. Five patients (4 TNFi and 1 nbDMARD) were reported to have renal involvement with biopsy results available in three patients (class IV lupus nephritis, immune complex glomerulopathy on infliximab and adalimumab, respectively; membranous glomerulonephritis in biological-naïve patient on methotrexate). Only 16.7% and 20% of events met ACR 1997 SLE classification criteria or SLICC 2012 classification criteria, respectively (table 4). Additional treatment following drug cessation such as steroids (IV or oral) or topical treatment (steroids and tacrolimus) was reported in 27 patients in the TNFi cohort (50%) and 1 patient in the nbDMARD cohort (20%).

**Table 4:** Descriptive characteristics of lupus and vasculitis-like events

Characteristics	nbDMARD cohort n (%)	TNFi cohort n (%)	Characteristics	nbDMARD cohort n (%)	TNFi cohort n (%)
**Lupus-like events**			**Vasculitis-like events**		
Limited to cutaneous manifestations only Cutaneous involvement (all) Malar rash Discoid rash Photosensitive rash SCLE rash Other† Missing description‡ Alopecia Mouth ulcers Constitutional symptoms Serositis (pericardial/pulmonary involvement) New arthralgia§ Haematological involvement Neurological involvement Renal involvement New ANA-positive Anti-dsDNA-positive Low complement (C3/C4) Antiphospholipid antibodies-positive ACR SLE criteria met SLICC SLE criteria met	3 (60) 4 (80) 2 (50) 1 (25) – 1 (25) – – – 1 (20) 1 (20) – 2 (40) – – 1 (20) 4 (80) 2 (40) – – 1 (20) 2 (40)	30 (55.5) 48 (89) 6 (12.5) 8 (16.7) 8 (16.7) 2 (4.1) 17 (35.4) 7 (14.6) 7 (13.0) 5 (9.2) 6 (11.1) 4 (7.4) 10 (18.5) 5 (9.3) 2 (3.7) 4 (7.4) 30 (55.6) 12 (22.2) 5 (9.3) 1 (1.9) 9 (16.7) 11 (20.0)	Limited to cutaneous manifestations only (included urticarial, bullous, purpuric and ulcerating lesions) *Systemic involvement Digital ischaemia Nailfold vasculitis Neurological involvement (small vessel vasculitis confirmed on sural biopsy; mononeuritis multiplex) Respiratory involvement (including cavitating lung lesions; pneumonitis and pulmonary emboli) ENT Renal involvement Ocular involvement (including temporal branch retinal vein occlusion of vasculitis type and episcleritis) Associated thromboembolism ANCA-positive Requiring IV methylprednisolone +/−cyclophosphamide	9 (64.3) 5 (35.7) 2 (14.3) – 1 (7.1) – – – 1 (7.1) – – 1 (7.1)	51 (63.0) 30 (37.0) 11 (13.6) 14 (17.3) 6 (7.4) 6 (7.4) 3 (3.7) 2 (2.5) 2 (2.5) 4 (4.9) 5 (6.2) 10 (12.3)

*Systemic involvement in VLE cases refers to extra-cutaneous involvement outlined below. In the VLE cases, ANCA status was not checked or reported in the majority of cases, with five patients with known positive status during the event (four patients pANCA-positive/MPO −ve and one patient cANCA-positive, PR3-positive).

†Other rashes included maculopapular, bullous, chilblain lupus rashes.

‡Patients with missing details regarding their cutaneous involvement were reported as ‘cutaneous lupus’ by the treating physician in conjunction with other lupus manifestations. Other positive serology detected in TNFi-treated patients with LLE included anti-Ro/La antibodies, antiribonucleoprotein (RNP) antibodies, perinuclear antineutrophil cytoplasmic antibodies (pANCA) positivity, antihistone antibodies in one patient each.

§No cases were classified as LLE solely on the basis of being ANA-positive and new arthralgia. All such cases developed arthralgia and other SLE manifestations concomitantly.

ACR, American College of Rheumatology; ANA, antinuclear antibodies; dsDNA, double-stranded DNA; nbDMARDs, non-biological disease-modifying antirheumatic drugs; ENT, ear, nose and throat;SCLE, subacute cutaneous lupus erythematosus; SLE, systemic lupus erythematosus; SLICC, Systemic Lupus International Collaborating Clinics; TNFi, tumour necrosis factor-α inhibitor.

Approximately two-thirds of cases in each cohort had VLE limited to the skin (table 4). Most common systemic manifestations included digital ischaemia, neurological involvement and respiratory involvement (table 4). Two patients on TNFi were reported to develop renal manifestations, one with renal vasculitis (no biopsy), the second with renal involvement, along with pneumonitis, new thrombus in aorta on CT angiogram and cANCA-positive status. Associated thromboembolic events were reported in four patients (4.9%). Systemic involvement was reported in 30/81 (37%) VLE patients on TNFi. The patient who developed cANCA/PR3-positive vasculitis died following the development of haemorrhagic alveolitis (with leukocytoclastic vasculitis on lung biopsy), new sinusitis and bilateral episcleritis while on adalimumab. However, systemic treatment with IV methylprednisolone+/−cyclophosphamide was required in the minority of patients treated with TNFi (n=10, 12.3%).

### Outcome following event

Of the 39 patients with LLE, outcomes included complete resolution (72%), partial resolution (+/−revision of diagnosis from RA to mixed connective tissue disease/SLE with on-going treatment) (15%), 1 death (2%) and unknown (11%). In patients who switched to a different biologic following LLE, there was a trend for some patients to have recurrent events on a second/third TNFi compared to switching to rituximab (see online supplementary figure S2). However, quantitative comparisons could not be made due to low numbers of subsequent events. There were three deaths reported in relation to VLEs.

## Discussion

This study is the first prospective observational study to specifically assess the risk of lupus and vasculitis events in patients with RA treated with TNFi. Although unadjusted estimates conferred an increased risk of LLE and VLE individually in TNFi-treated patients, following adjustment this no longer remained significant.

These data must be interpreted carefully with some clinically important points to take from these analyses. First, the absolute risk of LLE and VLE remains very small with an incidence rate of 10 and 15 per 10 000 pyrs of follow-up in the TNFi-treated cohort, respectively. Second, the majority of events was limited to cutaneous manifestations in both subsets, with resolution of symptoms (+/−treatment) in most of the LLE episodes. Third, there were inherent limitations common to observational studies. There are clear differences between the nbDMARD and TNFi-treated cohorts, the latter as expected and as per the national guidelines, have more severe disease at baseline, which may in turn be a risk factor for the events examined. Indeed, we found factors that reflect baseline disease severity were associated with higher rates of LLE and VLE. We addressed this issue of confounding by indication, by adjustment using a propensity score. The adjusted analysis of LLE and VLE separately did not demonstrate a significant risk attributable to the TNFi itself. It was not possible to distinguish if these cases would have occurred due to RA or TNFi treatment; however, the association of high-disease activity with VLE/LLE would suggest that uncontrolled disease may be responsible for triggering such events. While propensity scores address confounders present at baseline, they do not take into account time-varying confounders such as steroid use, disease activity and changing nbDMARD use or dose over the course of the study. Statistical techniques, such as marginal structural modelling, have been used to address time-varying confounders in RA previously;^22^ however, not used in this case as the required variables to generate weights were not measured at regular time points. Finally, while the comparator cohort of nbDMARD active disease patients is one of the strengths of the study, it is important to note the comparator includes exposure to drugs also associated with drug-induced lupus and vasculitis events. Therefore, while there may be no significant differences in adjusted risk when the two groups are compared, this may be due to comparison with a group of patients already at a higher risk of LLE/VLE. Such exposures may therefore introduce bias towards the null hypothesis. We accounted for this by performing sensitivity analyses, which excluded patients on putative drugs in both cohorts, which did not significantly change in the adjusted estimates.

To date, there have been few studies that were able to robustly assess drug-specific risk of these immune-mediated AEs. Such studies have not been adequately powered to definitively answer this question. In our study, infliximab appeared to carry the highest incidence rates of LLE and VLE, followed by etanercept and adalimumab. The highest risk observed with infliximab in the unadjusted analysis may be due to the fact that patients started on the drug were from a historical cohort and experienced more severe disease at the outset. However, after adjustment for confounders, 95% CIs overlapped. Prior studies have faced a number of limitations, including imprecise reported events from spontaneous pharmacovigilance, lack of an accurate denominator of TNFi-exposed patients and absence of an adequate comparator to assess rare outcomes such as immune-mediated AEs.^2^ ^8^ ^9^ ^13^ A French pharmacovigilance study recently attempted to examine drug-specific risk of LLEs from spontaneous reports, across all indications (including inflammatory bowel disease), and used a positive and negative control as comparators (known lupus-inducer isoniazid and paracetamol, respectively).^23^ In contrast to our study, infliximab and adalimumab were found to be associated with higher rates, with etanercept conferring a comparatively lower risk, although 95% CIs also overlapped with all three drugs. Similar to our study, of the 39 cases, few fulfilled SLE classification criteria. Other descriptive studies reported higher rates with infliximab and etanercept for LLE^2^ and VLE.^9^

LLE and VLE appeared to have a time-varying risk in the TNFi-treated cohort. The observation of greater risk of certain outcomes early in the disease course has been described previously with serious infections, including septic arthritis.^24–26^ For LLE/VLE, there may be a number of possible hypotheses to explain this risk pattern. First, it may be that TNFi patients have more severe disease at baseline (the latter associated with the outcome), which then improves with therapy therefore reflecting a reduction in risk. Second, it may indicate a depletion of susceptible individual's effect from the TNFi cohort, whereby patients who remain on the drugs are those who can tolerate them while those who are susceptible to the event select themselves out of the population at risk. Third, it may be that patients who are prone to developing LLE and VLE have a genetic predisposition to SLE/vasculitis, through shared genetic pathways common to RA and SLE for instance,^27^ and following a TNFi exposure ‘trigger’ develops the event early in the course of treatment, unblinding a condition that may have developed regardless. Fourth, it is possible that such events are associated with immunogenicity of the biologic, leading to secondary inefficacy (loss of response)^28–30^ and eventual switching to another biologic, which would mean such events occur early in the disease course. Finally, it may reflect a true reduction in risk of these events over time.

The strengths of this study are the well-characterised large sample size, systematic reporting of AEs from multiple sources (patients and clinicians) to improve validity, a comparator cohort that was simultaneously recruited and had active disease, making it as similar as possible to the TNFi arm. Missing baseline data were low and to minimise potential bias, multiple imputation was used. Additionally, classification of LLEs was attempted, as well as quantifying drug-specific risk of TNFi.

Although certolizumab patients are included in the analysis and no events were reported, inferences drawn from this are limited due to shorter follow-up and low numbers of recruited patients. Since we only included patients on first TNFi drug in the analysis, we excluded many patients on this drug who were switchers (including one patient who developed LLE). Patients on certolizumab had clear baseline differences to the rest of the TNFi cohort, including lower disease severity and higher concomitant nbDMARD use, which may also contribute to eventual lower rates. We also excluded patients with baseline SLE and systemic vasculitis; therefore, these results cannot be extrapolated to patients with overlap of these conditions. ANA was not consistently measured or reported in patients at baseline; therefore, it was not possible to assess if the presence/emergence of ANA affects future risk of clinical events. While certain VLEs, such as digital ischaemia and serious thrombotic events, were observed in our study (and previously associated with antidrug antibody formation^7^), our patients did not have serum samples collected; therefore, evaluation of immunogenicity (or antiphospholipid antibodies where status was absent) could not be performed.

## Conclusions

The increased risk in the TNFi group of LLE and VLE was not significant after full adjustment of baseline covariates, suggesting no increased risk following adjustment for confounding by indication. The addition of TNFi to nbDMARD, therefore, does not alter the risk of either event in patients with RA selected for TNFi. The absolute risk of LLE and VLE remains low. The risk of both events was time-varying and highest in the first year of treatment. Clinicians should be aware of these rare but potentially important events in TNFi-treated patients, especially as their presentation may not fulfil usual classification criteria at the outset.
