# Correction: *Drug-specific risk and characteristics of lupus and vasculitis-like events in patients with rheumatoid arthritis treated with TNFi: results from BSRBR-RA*

**DOI:** 10.1136/rmdopen-2016-000314corr1

**Published:** 2017-02-23

Jani M, Dixon WG, Kersley-Fleet L, *et al*. Drug-specific risk and characteristics of lupus and vasculitis-like events in patients with rheumatoid arthritis treated with TNFi: results from BSRBR-RA. *RMD Open* 2017;3:e000314.

The surname of the third author of this paper was spelt incorrectly. The correct surname is “Kearsley-Fleet”. 

