# Supplementary material for: Drug-specific risk and characteristics of lupus and vasculitis-like events in patients with rheumatoid arthritis treated with TNFi: results from BSRBR-RA
Source: RMD Open. 2017 Jan 17;3(1):e000314. doi: 10.1136/rmdopen-2016-000314 (PMC5255894; doi:10.1136/rmdopen-2016-000314)
Supplement: supplementary data [file rmdopen-2016-000314supp.pdf]

Supplementary material

Table S1: Dubois' Guidelines for Diagnosis of Drug-Induced Lupus

**1. Continuous treatment with a known lupus-inducing** drug for ≥ 1month and usually much longer
**2. Presenting symptoms:**<br>**Common**- arthralgia, myalgias, malaise, fever, serositis, polyarthritis<br>**Rare**- rash or other associated dermatological problems, glomerulonephritis
**3. Unrelated symptoms suggestive of SLE:**<br>Multisystem involvement especially neurological, renal and skin symptoms
**4. Laboratory profile**<br>**Common:** ANA which is as a result of anti-histone antibodies especially IgG anti-[(H2A-H2B)-DNA], leukopenia, thrombocytopenia and mild anaemia, increased erythrocyte sedimentation rate<br>**Rare:** Antibodies to native DNA, Sm, RNP, SS-A/Ro, SS-B/La, hypocomplementemia
**5. Improvement and permanent resolution of symptoms** generally within days or weeks after discontinuation of therapy. Serological findings, especially autoantibody levels may take months to resolve.

Reproduced with permission of the copyright owner. Dubois' Lupus Erythematosus and Related Syndromes 8th edition - ISBN: 9781437718935. Patients were classified as having a lupus-like event for the purposes of this analysis if the met ≥ 2 of the above criteria.

Figure S1: Selection of patients for analysis

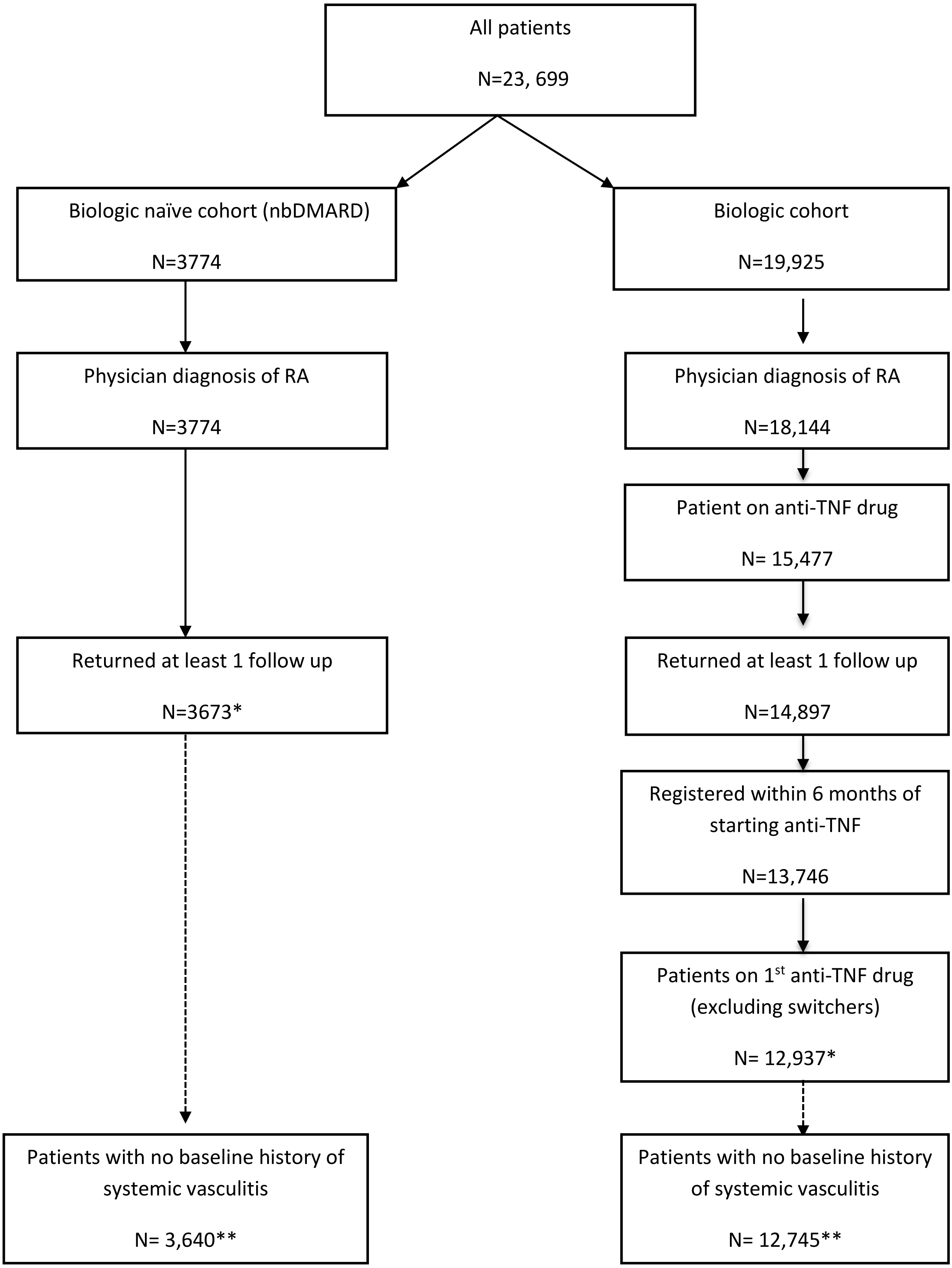

*Denominator for lupus-like events analysis **Denominator for vasculitis-like events and combined immune mediated adverse events analyses. Abbreviations: anti-TNF, anti-tumour necrosis factor; nbDMARD, non-biologic disease modifying anti-rheumatic drug; RA, rheumatoid arthritis.

Statistical methodology supplementary information

Missing data

Multiple imputations were performed in Stata version 13.1 using the ICE command. Missing data were present in the following baseline variables: disease duration, baseline HAQ, baseline HAQ score, smoking and ethnicity. The imputation model was constructed separately for the nbDMARD and anti-TNF cohorts. Age, gender, disease duration, baseline HAQ, separate components of baseline DAS28 score (swollen joint count, tender joint count, patient global visual analogue score and ESR), rheumatoid factor positivity, co-morbidity, smoking status, ethnicity, entry year and baseline steroid exposure were included as predictors within the imputation model. Twenty imputation cycles were performed and final data analysed using Rubin's rule with the MIM command.

Table S2: Missing baseline data

**Variable; n missing (%)**	**nbDMARD** (n=3,673)	**TNFi** (n=12,937)
Disease duration	23 (1)	131 (1)
HAQ score	729 (20)	805 (6)
DAS28 score	55 (1)	102 (1)
Smoking	18 (0)	128 (1)
Ethnicity	648 (18)	2008 (16)
Rheumatoid factor positive	3 (0)	118 (1)

Abbreviations: HAQ, Health assessment questionnaire; DAS 28, Disease activity score of 28 joints

Propensity Scores

Confounders were adjusted using an inverse probability of treatment weighting score to assess drug-specific differences accurately. Logistic regression was used to generate a probability of treatment (propensity) score. The final propensity score for vasculitis-like events included age, gender, disease duration, baseline HAQ scores, baseline DAS28 score, a composite measure for comorbidities, rheumatoid factor positive status, year of recruitment, baseline nbDMARD use, baseline oral steroid. For the lupus-like events and combined-event analysis, ethnicity (dichotomised as white/non-white) was also included. The inverse of the probability (1 minus the inverse of the probability in the nbDMARD cohort) was used as the treatment weight in the analysis. Weights greater than 20 were truncated in the final combined-events analysis to avoid de-stabilising the model. The balance of the variables was tested between the nbDMARD and anti-TNF cohorts using standardised differences rather than estimated bias.

Table S3: Sensitivity analysis following exclusion of secondary causes leading to event

**Lupus like events (excluding all patients with known lupus inducing drugs)†**						
	**nbDMARD (n=2,083)**	**All TNFi (n=9,625)**	**Etanercept (n=3,513)**	**Adalimumab (n=3,038)**	**Infliximab (n=2,658)**	**Certolizumab (n=416)**
**Total follow up time (patient-years)**	**10,070**	**39,521**	**16,580**	**11,844**	**10,472**	**625**
**Number**	**3**	**44**	**19**	**8**	**17**	**0**
**Crude incidence rate of lupus like event per 10,000 person-years (95% CI)**	**2.8 (0.9, 8.8)**	**11.0 (8.2, 14.7)**	**11.3 (7.2, 17.7)**	**6.6 (3.3, 13.3)**	**16.1 (10.0, 25.8)**	**-**
**Unadjusted hazard ratio (95%-CI)**	**Referent**	**3.71 (1.15,12.0)***	**4.06 (1.20, 13.73)**	**2.20 (0.58, 8.30)**	**5.31 (1.55, 18.12)***	**-**
**Propensity-adjusted hazard ratio (95%-CI)**	**Referent**	**2.33 (0.56, 9.71)**	**1.93 (0.48, 7.67)**	**2.62 (0.40, 17.11)**	**2.53 (0.62, 10.27)**	**-**

**Vasculitis like events (excluding events with a possible secondary trigger) ††**						
	**nbDMARD (n=3,567)**	**All TNFi (n=12,489)**	**Etanercept (n=4,351)**	**Adalimumab (n=4,233)**	**Infliximab (n=3,216)**	**Certolizumab (n=689)**
**Total follow up time (patient-years)**	**20,148**	**51,530**	**20,936**	**16,886**	**12,676**	**1032**

**Number**	**13**	**68**	**29**	**17**	**22**	**0**
**Crude incidence rate of lupus like event per 10,000 person-years (95% CI)**	**6.5 (3.7, 11.1)**	**13.2 (10.4, 16.7)**	**(9.6, 20.0)**	**10.1 (6.3, 16.2)**	**17.4 (11.4, 26.3)**	**-**
**Unadjusted hazard ratio (95%-CI)**	**Referent**	**1.92 (1.06, 3.47)***	**2.17 (1.12, 4.18)***	**1.41 (0.68, 2.90)**	**2.47 (1.24, 4.92)***	**-**
**Propensity-adjusted hazard ratio (95%-CI)**	**Referent**	**1.05 (0.32, 3.45)**	**1.34 (0.40, 4.50)**	**0.66 (0.18, 2.36)**	**1.28 (0.37, 4.50)**	**-**

* $p<0.05$. †Baseline use of the following drugs, lead to exclusion of patients in the LLE sensitivity analysis: quinidine, isoniazid, chlorpromazine, hydralazine, methylodopa, carbimazole, penicillin, propylthiouracil, phenobarbitone, phenytoin, diltiazem, lithium; baseline/current use of sulfasalazine, leflunomide, minocycline and penicillamine. †† Baseline use of the following drugs, lead to exclusion of patients in the vasculitis sensitivity analysis: propylthiouracil, carbamazepine, phenobarbitone, phenytoin, hydralazine, sodium valproate; baseline/current use of minocycline and penicillamine. In addition patients with a likely secondary cause of vasculitis (e.g. infection) and recorded nailfold vasculitis at baseline were also excluded. Abbreviations: CI, confidence interval; nbDMARDs, non-biological disease-modifying anti-rheumatic drugs; TNFi tumour necrosis factor-alpha inhibitor.

Figure S2: Outcome of switching to biologics following lupus-like events

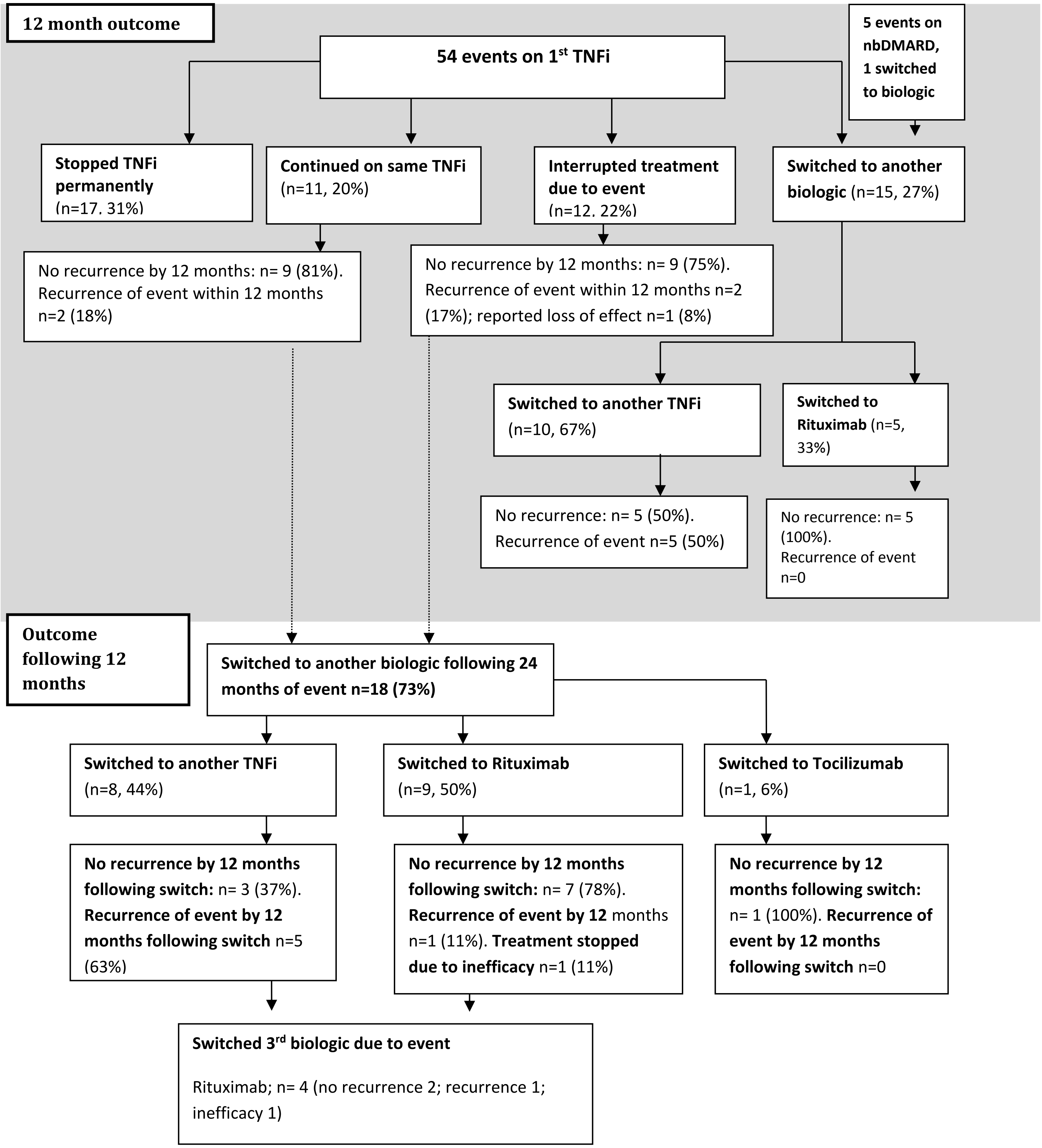

Of the 4 nbDMARD patients who did not switch to a biologic, 1 patient switched to methotrexate from penicillamine, 1 patient continued with azathioprine for 2 years followed by death (unrelated to LLE), 1 patient continued with methotrexate and hydroxychloroquine and was deemed to have SLE following the initial event and 1 patient stopped their methotrexate for a few years post event and was restarted when the joint disease started to flare. Abbreviations: TNFi, tumour necrosis-factor inhibitor

Table S4 MedDRA preferred terms used to search for Lupus Like Events and Vasculitis Like Events

Lupus Like Events	Vasculitis Like Events
Cutaneous lupus erythematosus	Vasculitis
Systemic lupus erythematosus/ SLE	Vasculopathy
Lupus-like syndrome	Purpuric vasculitic rash
Lupus nephritis	Vasculitic ulcers
Systemic lupus erythematosus rash	Vasculitis lesions
Drug induced lupus	Digital Vasculitis
	Drug induced vasculitis

104 and 217 events for LLE and VLE respectively were identified using MedDRA codes as listed above and a free text search within the adverse event reported fields that included the terms “lupus” and “vasculitis” like events. Cases were screened and excluded if they did not meet eligibility criteria outlined in the methods section.
